# Sphaeromyxids form part of a diverse group of myxosporeans infecting the hepatic biliary systems of a wide range of host organisms

**DOI:** 10.1186/1756-3305-6-51

**Published:** 2013-03-01

**Authors:** Árni Kristmundsson, Mark A Freeman

**Affiliations:** 1 Institute for Experimental Pathology, University of Iceland, Keldur v/Vesturlandsveg, Reykjavik, 112, Iceland; 2 Institute of Ocean and Earth Sciences, University of Malaya, Kuala Lumpur, 50603, Malaysia

**Keywords:** Sphaeromyxa, Lycodes, Gall bladder, Myxosporean, Myxidium, Hepatic biliary group, Chloromyxum

## Abstract

**Background:**

Approximately 40 species of *Sphaeromyxa* have been described, all of which are coelozoic parasites from gall bladders of marine fish. They are unique amongst the myxosporeans as they have polar filaments that are flat and folded instead of being tubular and spirally wound. This unusual feature was used as a subordinal character to erect the suborder Sphaeromyxina, which contains one family, the Sphaeromyxidae, and a single genus *Sphaeromyxa.*

**Methods:**

In the present study, we examine eelpout from the genus *Lycodes* from Iceland for the presence of myxosporean parasites in the gall bladder and perform morphological and DNA studies.

**Results:**

A novel myxosporean, *Sphaeromyxa lycodi* n. sp., was identified in the gall bladders of five of the six species of *Lycodes* examined, with a prevalence ranging from 29 - 100%. The coelozoic plasmodia are large, polysporous and contain disporic pansporoblasts and mature spores which are arcuate. The pyriform polar capsules encase long and irregularly folded ribbon-like polar filaments. Each spore valve has two distinct ends and an almost 180° twist along the relatively indistinct suture line. The single sporoplasm is granular with two nuclei. *Sphaeromyxa lycodi* is phylogenetically related to other arcuate sphaeromyxids and is reproducibly placed with all known sphaeromyxids and forms part of a robustly supported clade of numerous myxosporean genera which infect the hepatic biliary systems of a wide range of hosts.

**Conclusions:**

*Sphaeromyxa lycodi* is a common gall bladder myxosporean in eelpout of the genus *Lycodes* from Northern Iceland. It has characteristics typical of the genus and develops arcuate spores. Molecular phylogenetic analyses confirm that sphaeromyxids form a monophyletic group, subdivided into straight and arcuate spore forms, within the hepatic biliary clade that infect a wide range of freshwater associated animals. The ancestral spore form for the hepatic biliary clade was probably a *Chloromyxum* morphotype; however, sphaeromyxids have more recently evolved from an ancestor with a spindle-shaped *Myxidium* spore form. We recommend that the suborder Sphaeromyxina is suppressed; however, we retain the family Sphaeromyxidae and place it in the suborder Variisporina.

## Background

Myxosporeans are common parasites of fish and have a two-host lifecycle involving an invertebrate that is generally an annelid worm. The vertebrate host is typically a fish but other aquatic-associated vertebrates such as turtles, waterfowl and amphibians as well as terrestrial insectivorous mammals are also reported as hosts [1-5]. There are approximately 40 species described from the genus *Sphaeromyxa* Thélohan 1892, all of which are coelozoic parasites in gall bladders of marine fish and form characteristic large flat plasmodia. Although not usually associated with serious pathology, some may cause blockages of bile ducts which results in bile accumulation and liver inflammation [6]. Species of this genus are unusual in that they do not have a typical tube-like polar filament that is spirally wound in the polar capsule. Rather, it is flat in section, broad at the base, gradually tapering along its length and is folded upon itself several times in the polar capsule. Lom and Noble [7] proposed this unusual feature as a new subordinal character and erected the suborder Sphaeromyxina Lom et Noble, 1984 to include a single new family Sphaeromyxidae Lom et Noble, 1984. In Thélohan’s original description of the genus, *Sphaeromyxa,* he considered it to be a member of the family Myxidiidae Thelohan 1892. DNA sequence data for sphaeromyxids are somewhat limited, with information available for only 5 species. However, currently they are one of the few monophyletic myxosporean taxa [8] and unusually group with a range of other myxosporean genera that infect the gall bladders of freshwater hosts [9].

There are few reports of myxosporeans from eelpout (Zoarcidae). The type species of the genus *Shulmania*, *S. ovale*, was described from the urinary bladder of *Lycodes esmarkii* from the Canadian Atlantic [10]. In the Pacific, *Myxidium melanostigmum* was described from the gall bladder of the eelpout *Melanostigma pammelas* a deepwater fish off the Californian coast [11] and *Myxobolus aeglefini* was found in the skeletal muscle of porous-head eelpout *Allolepis hollandi* from the Sea of Japan [12].

In the present study, we examine eelpout, from the genus *Lycodes*, from Iceland for the presence of myxosporean parasites in the gall bladder.

## Methods

**Figure 1:**
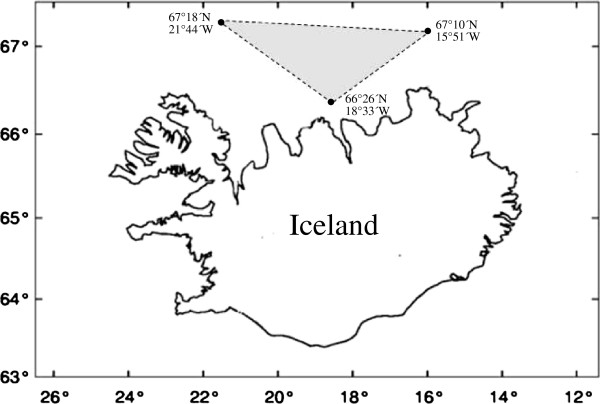
**Sampling area of *Lycodes *spp. north of Iceland (shaded area) demarcated by three coordinates.**

Fish were sampled by trawling (300 – 500 m) north of Iceland in July 2012 during an annual survey performed by the Marine Research Institute in Iceland (Figure 1). These included adult fish of *Lycodes esmarkii* (length range 43 – 62 cm: n = 10), *L. gracili*s (18 – 27 cm; n = 21), *L. reticulatus* (11 – 23 cm; n = 22), *L. pallidus* (18 – 26 cm; n = 4), *L. seminudus* (16 – 36 cm; n = 10) and *L. eudipleurostictus* (10 – 29 cm; n = 22). Immediately after catching, the fish were frozen aboard the research vessel. After the survey the fish were taken to a laboratory and kept frozen until examination.

### Fresh material - spore measurements

Thawed fish were dissected, their gall bladder removed and a drop of its contents put on a microscopic slide and screened for the presence of myxosporean infections at a magnifications of 200× - 400×. Initially two species of fish were chosen, *L. reticulatus* and *L. eudipleurostictus*, and descriptions and measurements of spores were taken following the guidelines of Lom and Arthur [13]. Fresh spores were measured and photographed using bright field and Nomarski illumination at magnification up to 1250×. All other fish species were checked for the presence of myxosporeans and samples taken for DNA analyses.

### Histology

Gall bladders from two infected fish, one *L. reticulatus* and one *L. eudipleurostictus,* were fixed in 10% buffered formalin, embedded in paraffin wax, sectioned (4 μm), stained with Giemsa and Haematoxylin and Eosin and prepared for histological examination according to routine protocols. In addition, air dried smears from infected gall bladders were fixed in methanol and stained with Giemsa and Haematoxylin and Eosin.

### SEM methods

The contents of an infected gall bladder from each species (*L. reticulatus* and *L. eudipleurostictus*) of host fish were fixed in 2.5% glutaraldehyde for 4 hrs at 4°C, and then rinsed four times in 100 mM sodium cacodylate buffer pH 7.2 allowing the spores to settle under gravity between each rinse. The resulting spore suspension was passed through a 0.4 μm Whatman Cyclopore® track-etched polycarbonate membrane using a syringe and filter clamp. The membrane was then post-fixed in 1% osmium tetroxide in 100 mM sodium cacodylate buffer pH 7.2 for 2hrs and taken through an ethanol series of 30%, 60%, 90% and 2 × 100% 30 mins each, transferred into 50% hexamethyldisilazane (HMDS) in 100% ethanol followed by two changes of 100% HMDS each for 45 min. Excess HMDS was removed and the membranes allowed to air dry overnight. The membranes were then mounted onto aluminium stubs, earthed with silver dag paint and sputter-coated with gold. Samples were viewed with a Jeol JSM 6460 LV SEM instrument.

### DNA analysis

Gall bladder contents from three infected fish from, *L. reticulatus* and *L. eudipleurostictus*, were used in initial DNA extractions and to obtain the majority (18e-18gM) of the small subunit ribosomal DNA (SSU rDNA) sequence. Total DNA was extracted using a GeneMATRIX kit (EURx Poland) following the tissue protocol. Parasite SSU rDNA was amplified using the myxosporean PCR primers and methodology described by Freeman *et al.*[14] and the additional primers set 390f 5’agagggagcctgagaaacg 3’ and 1830r 5’ tctaagggcatcacagacctg 3’ using the same PCR conditions. DNA samples from additional infected fish, 2 per species, *L. gracilis*, *L. pallidus* and *L. seminudus*, were extracted as above and amplified using primers designed to be specific for sphaeromyxid taxa, Sphy-F 5’gaaaggctcagtatatcag 3’ and Sphy-R 5’ tattcaaggcacgyyatgc 3’ which amplify a 744 base pair region of the SSU rDNA that includes the phylogenetically informative V4 region. PCR conditions were the same as those described above. All PCRs were completed in triplicate and PCR products of the expected sizes were recovered using a GeneMATRIX PCR products extraction kit (EURx Poland).

Sequencing reactions were performed using BigDyeTM Terminator Cycle Sequencing chemistry utilising the same oligonucleotide primers that were used for the original PCRs. DNA sequencing was performed in both forward and reverse directions for all PCR products and nucleotide BLAST searches performed for each sequence read to confirm a myxosporean origin [15]. The contiguous sequence was obtained manually using CLUSTAL_X and BioEdit [16,17]. CLUSTAL X was used for the initial sequence alignments of 54 myxosporean taxa, with the settings for gap opening/extension penalties being adjusted manually to achieve optimum alignments. The final alignment was manually edited using the BioEdit sequence alignment editor and contained 2619 characters of which 1358 were informative sites.

Phylogenetic analyses were performed using the maximum likelihood methodology in PhyML [18] with the general time-reversible substitution model selected and 100 bootstrap repeats. Maximum parsimony in PAUP*4.0 beta10 [19] using a heuristic search with random taxa addition (10 replications), the ACCTRAN-option, and the TBR swapping algorithm with gaps treated as missing data and branch supports obtained with 1000 bootstrap replicates. Bayesian inference (BI) analysis using MrBayes v. 3.2.1 [20]. The BI analysis models of nucleotide substitution were first evaluated for the alignment using MrModeltest v. 2.2 [21]. The most parameter-rich evolutionary model based on the AIC was the general time-reversible, GTR+I+G model of evolution. Therefore, the settings used for the analysis were nst = 6, with the gamma- distributed rate variation across sites and a proportion of invariable sites (rates = invgamma). The priors on state frequency were left at the default setting (Prset statefreqpr = dirichlet (1, 1, 1, 1)). Posterior probability distributions were generated using the Markov Chain Monte Carlo (MCMC) method with four chains being run simultaneously for 1000,000 generations. Burn in was set at 2500 and trees were sampled every 100 generations making a total of 7500 trees used to compile the majority rule consensus trees.

#### *S. lycodi*

In accordance with section 8.6 of the ICZN's International Code of Zoological Nomenclature, details of the new species have been submitted to ZooBank with the Life Science Identifier (LSID) zoobank.org:pub:B7DFC3E9-5F5A-4239-B811-4C435DAA5423.

## Results

Five of six *Lycodes* species examined were found to be infected with a novel *Sphaeromyxa* species. The prevalence figures for each fish species were: *Lycodes pallidus*: number infected/total number examined = 4/4; prevalence = 100%, *L. reticulatus*: 18/22; 82%, *L. seminudus*: 7/10; 70%, *L. eudipleurostictus*: 15/22; 68%, *L. gracilis*: 6/21; 29%, *L. esmarkii*: 0/10; 0%.

### Description of *Sphaeromyxa lycodi* n. sp

**Figure 2:**
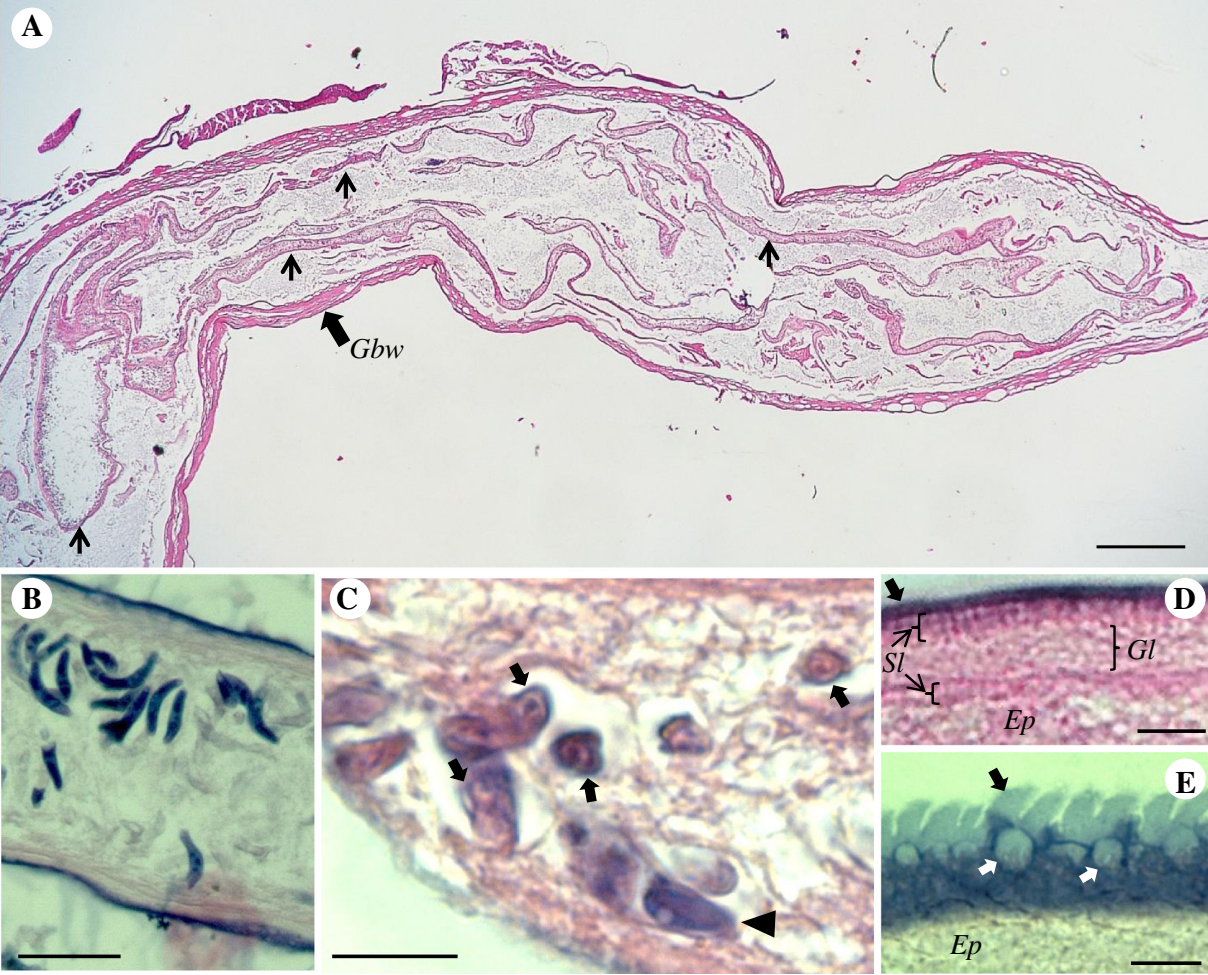
**Stained histological sections.** (**A**) A section through a gall bladder infected with *Sphaeromyxa locodi* n. sp. showing large polymorphic plasmodia (thin arrows) occupying large parts of its volume. (**B**) Mature *S. lycodi* spores within a plasmodium. (**C**) Several developing pansporoblasts (arrows) and one mature spore (arrowhead) within a plasmodium. (**D**) Typical ectoplasm of a plasmodium; a narrow compact outer layer (broad arrow), a triple eosinophilic inner layer, a broader finely granular layer (*Gl*) enclosed by two radially striated layers (*Sl*). (**E**) A less commonly detected ectoplasmic layer with spherical bodies (white arrows) and villar protrusions (black arrow). (**A**), (**C**) and (**D**) are stained with Haematoxylin and Eosin stain but (**B**) and (**E**) with Giemsa. Scale bars: (**A**) = 500 μm; (**B**) = 30 μm (**C**) = 10 μm; (**D**) and (**E**) = 5μm. *Gbw* - gall bladder wall. *Ep* – endoplasm.

**Figure 3:**
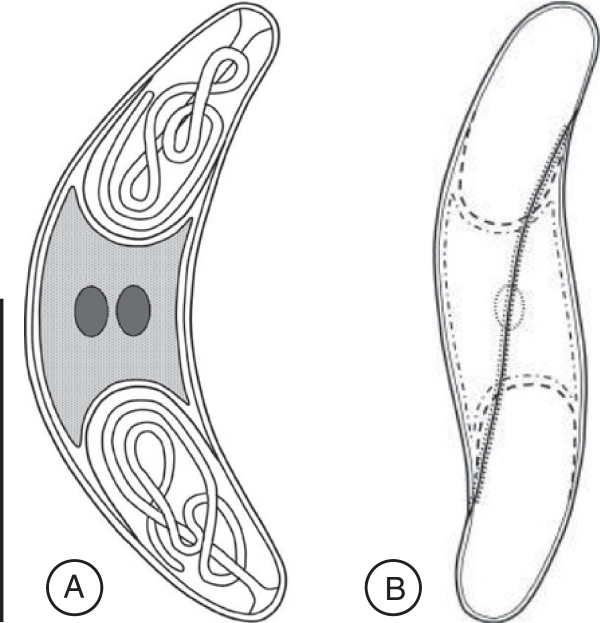
**Line drawings of a mature spore of *Sphaeromyxa lycodi *n. sp., (A) Frontal view and (B) sutural view. Scale bar = 10 μm.**

**Figure 4:**
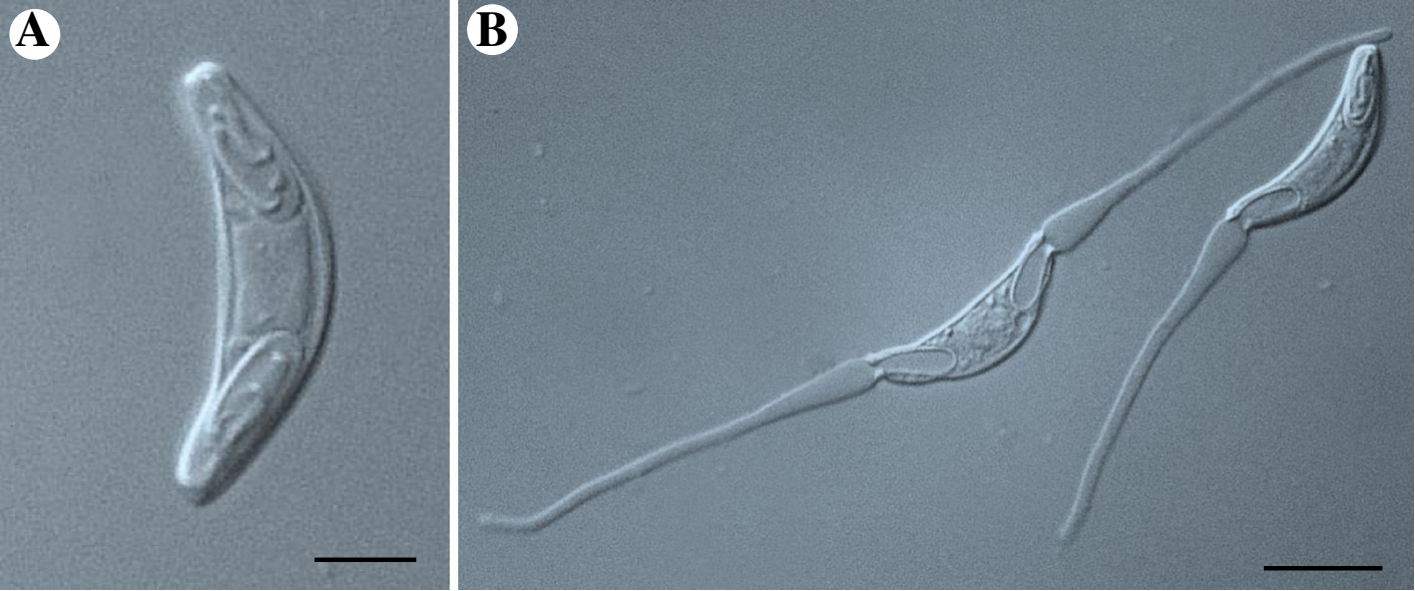
**Micrograph of fresh mature spores of *Sphaeromyxa lycodi *n. sp. (Nomarski interference).** (**A**) Frontal view with its polar filament folded in the polar capsules. (**B**) Two mature spores with their polar filaments extruded; 1.5 - 2 times longer than the valve length. Note the morphology of the polar filaments; broad at the base and tapering towards the ends. Scale bars: (**A**) = 5 μm, (**B**) = 10 μm.

The plasmodia are coelozoic, i.e. floating freely in the bile of the gall bladder and commonly occupying significant parts of the gall bladder’s volume, causing opacity in some cases. They are polymorphic, long and slender with irregular and long pseudopodial projections (Figure 2A). They are wrapped around themselves as well as neighbouring plasmodia; similar to rivets of tangled yarn threads. Estimating their exact length is problematic but the longest unbroken plasmodium detected in histological sections was approximately 10 mm long. They are polysporous and packed with numerous mature spores and developing sporoblasts (Figures 2B, C). They form disporic pansporoblasts. Most commonly the ectoplasm is composed of a narrow (1.0 - 1.5 μm) compact outer layer and a thicker (5 - 7 μm) and triple eosinophilic inner layer; a finely granular layer enclosed with radially striated layers (Figure 2D). Occasionally, plasmodia with ectoplasm having spherical bodies and prominent villar projections were detected (Figure 2E). The endoplasm is vacuolated and loosely connected. In frontal view the spore body is arcuate and tapers towards its rounded ends. In sutural view the spores are slightly sigmoid, tapering somewhat towards the blunt ends. The sporoplasm is granular with a pair of ovoid and centrally located nuclei parallel along the shorter axis. The pyriform polar capsules encase long and irregularly folded polar filaments. When extruded, the filaments appear flat along their entire length, broad where they exit the spore valve and gradually taper along their length (Figures 3A, B and 4A, B).

**Figure 5:**
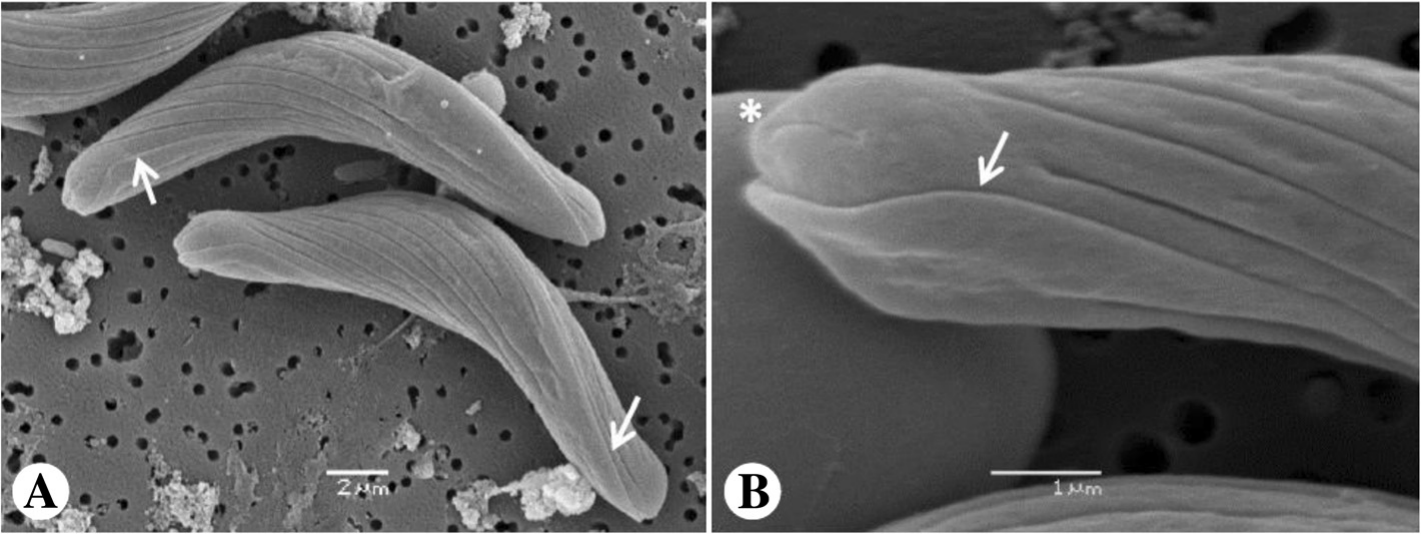
**Scanning electron micrographs of *Sphaeromyxa lycodi *n. sp. Spores have an indistinct suture line (white arrows).** Each valve has one rounded/bulbous end that supports the polar capsule and the other more spoon-like to receive the polar capsule from the opposing valve. The valves have an almost 180° twist along the suture length so that like ends appear to be in the same plane. Striations are present that start from the rounded/bulbous end extending along the valve but not for the entire length. There is a terminal short striation on the rounded/bulbous ends (white asterisk).

**Figure 6:**
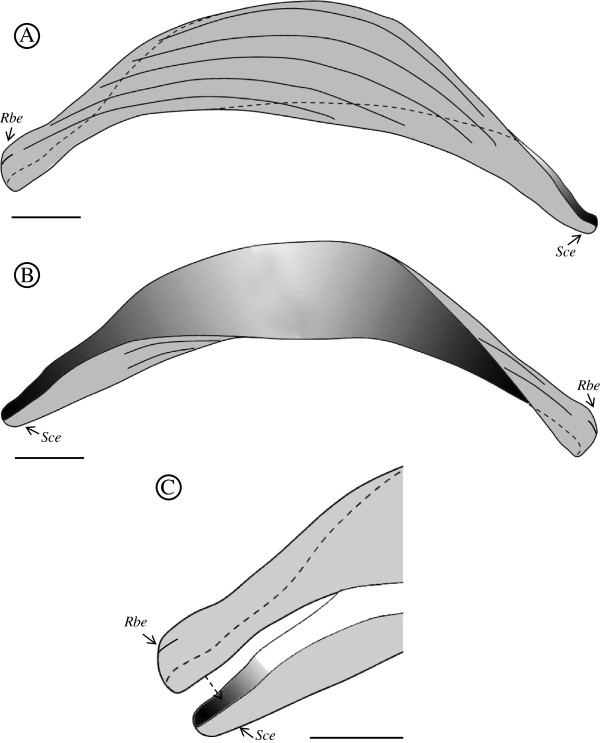
**(A) and (B) Line drawings showing the frontal view of the two valves of *S. lycodi *n. sp. separated.** Each valve has two different ends; a round/bulbous shaped end (Rbe) and a spoon/cup shaped end (Sce). The valves have an approximately 180° twist and consequently analogous ends of the two valves lie in the same plane. In frontal view most of the one valve's body is visible (**A**) but only the ends of the opposing valve (**B**). (**C**) The suture of the valve ends. The Rbe type appears to sit inside the Sce end and supports the polar capsule. Scale bars = 2 μm.

The suture line is relatively indistinct and similar in appearance to the valve striations, of which there are 6 – 7 on each spore valve. Each valve has one bulbous / rounded end that houses the polar capsule with the other end more spoon-like to receive the polar capsule from the opposing rounded valve end. The valves have an almost 180° twist along the suture length so that like ends appear to be in the same plane. Striations are present that start from the rounded end and extend down the valve, parallel to the suture, but not along the entire length. There is a short terminal striation on the rounded ends (Figures 5A, B and 6A, B).

Dimensions, based on measurement of 60 spores (20 for spore thickness and length of polar filaments) are as follows. Spore length (straight distance between the tips of the arcuate spore) 19.6 – 25.3 μm (mean ± s.d. = 22.4 ± 1.4), spore width 4.6 – 6.9 μm (5.7 ± 0.6), spore thickness (sutural view) 4.5 – 6.2 μm (5.7 ± 0.8), length of polar capsule 5.8 – 9.8 μm (8.32 ± 1.0), width of polar capsule 2.5 – 4.5 μm (3.3 ± 0.8), length of polar filaments 28.8 – 48.3 μm (36.5 ± 5.6). Spore measurements of *S. lycodi* from *L. reticulatus* and *L. eudipleurostictus* were found to be comparable in all dimensions, and were not significantly different.

### DNA analysis

The same myxosporean SSU rDNA sequence was obtained from both *L. reticulatus* and *L. eudipleurostictus*, with a contiguous sequence of 1983 bp submitted to Genbank under the accession number KC524734. Blast searches revealed the closest match in the databases to be *Sphaeromyxa kenti* and isolates of *Sphaeromyxa hellandi*, with a 94% and 91% identity respectively. Shorter sequenced regions of the SSU rDNA from *L. gracili*s, *L. pallidus* and *L. seminudus* all had identical sequence reads to the two type species with longer reads.

**Figure 7:**
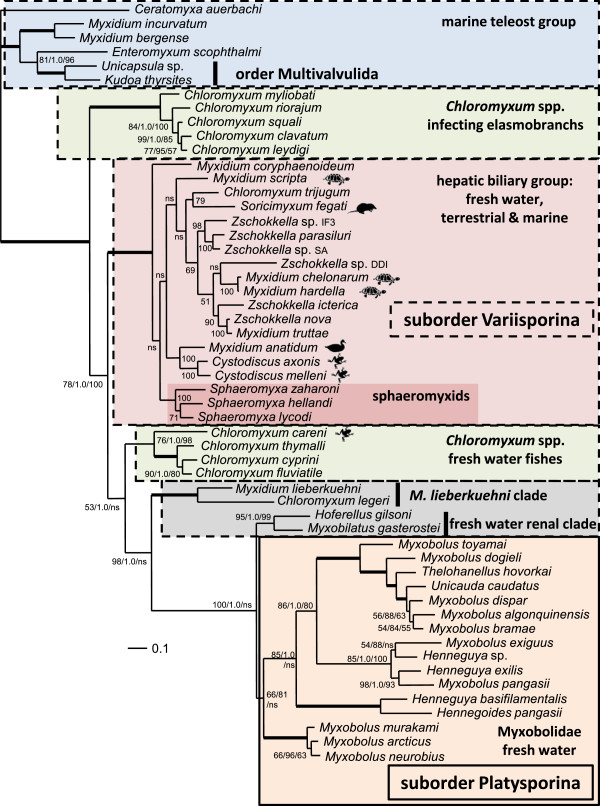
**Maximum likelihood topology based on dataset of 54 aligned myxosporean SSU rDNA sequences, generated using the general time reversible model of nucleotide substitution in PhyML.** Thick branches terminate in a node that received full support from three independent phylogenetic methodologies, numbers at the nodes refer to bootstrap support values for maximum likelihood (100 samplings), Bayesian posterior probability support and percentage bootstrap support for maximum parsimony (1000 samplings), (ns) indicates an unsupported node or one with a support value below 50. The light red shaded box represents a well-supported clade of myxosporeans that infect the hepatic biliary systems of a wide range of host organisms, the number at the nodes in this clade refer to maximum likelihood support values (see Figure 8 for Bayesian and maximum parsimony topologies for this clade). The darker red shaded box within the hepatic biliary clade contains *Sphaeromyxa lycodi* and other sphaeromyxid taxa. The shaded areas bordered by a bold dashed line represent taxa from the suborder Variisporina, with the exception of the Multivalvulida sequences from the marine teleost group (blue box).The light orange shaded area, bordered by a solid line contains taxa from the suborder Platysporina. All myxosporean sequences were taken from fish hosts unless specified with symbols after the specific names: representing turtle, shrew, waterfowl or amphibian hosts.

**Figure 8:**
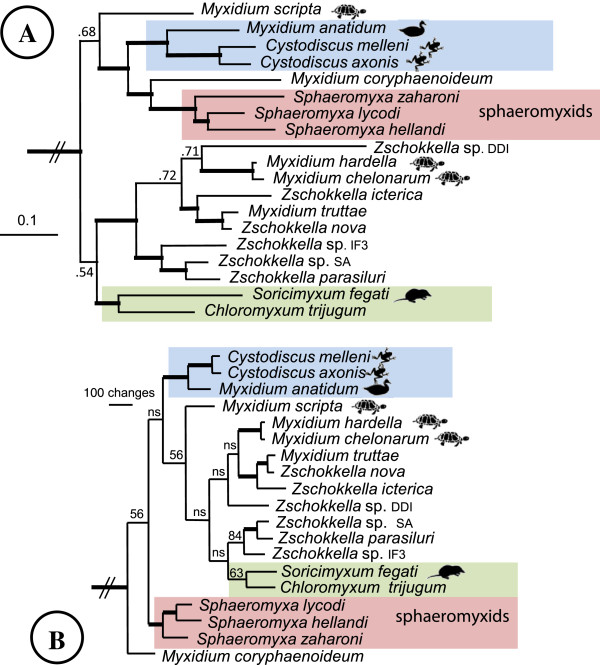
**Part of the phylogenetic trees for Bayesian analysis (A) and maximum parsimony analysis (B) for the nineteen myxosporean taxa that form the hepatic biliary clade; taken from trees generated using the same alignment of 54 taxa used in Figure**7**.** Thick branches represent a support value of >95 and (ns) indicates nodes with a support of <50. *Sphaeromyxa lycodi* is strongly supported in a clade with other sphaeromyxid taxa and has *Myxidium coryphaenoideum* as the closest known relative in both trees, but receiving very strong support in the Bayesian analysis. Shaded boxes represent clades that were recovered in all analyses.

Phylogenetic analyses using three methodologies produced congruent tree topologies with respect to the positioning and members of the major clades (Figure 7). *Sphaeromyxa lycodi* was reproducibly placed with other sphaeromyxid taxa in all analyses and formed part of a robustly supported clade of 19 taxa that are found infecting the hepatic biliary systems of a wide range of hosts (Figure 7). Although the hepatic biliary clade was strongly supported in all analyses, the relative positions of the 19 taxa varied depending on the phylogenetic methodology used (Figures 8A, B). Nevertheless, the sphaeromyxids were always robustly placed in a monophyletic group and were most closely related to *Myxidium coryphaenoideum* in all analyses. In both maximum likelihood and maximum parsimony topologies *M. coryphaenoideum* was the basal taxon for the hepatic biliary clade (Figures 7 and 8B), however, in the Bayesian analysis *M. coryphaenoideum*, together with the sphaeromyxids, formed a sister clade to one containing *Myxidium anatidum* and *Cystodiscus* spp. infecting waterfowl and amphibians respectively (Figures 8A). *Myxidium hardella* and *M. chelonarum*, from freshwater turtles, consistently grouped together in all analyses and formed as a sister clade to the fish-infecting species. However, the position of *Myxidium scripta*, also from freshwater turtles, in the group was not consistent and was poorly supported in all tree topologies and did not group with other species infecting turtles. *Soricimyxum fegati* isolated from the liver of the common shrew formed a consistent clade in all analyses with *Chloromyxum trijugum* infecting sunfish gall bladder and formed as a sister clade to the fish-infecting group (Figures 7 and 8A, B).

**Figure 9:**
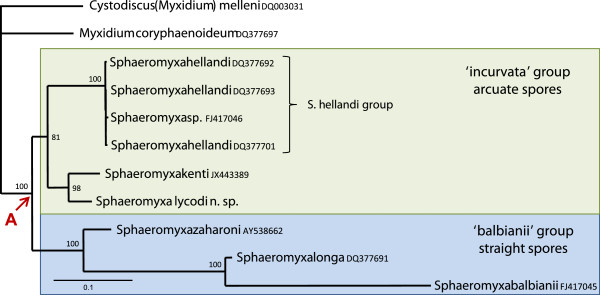
**Maximum likelihood phylogeny based on 11 SSU rDNA sequences (2033 characters) of sphaeromyxids and related taxa.***Sphaeromyxa lycodi* forms a robust clade with *S. kenti*, which is a well-supported sister clade to the *S. hellandi* group. The sphaeromyxids form two robustly supported groups from node A (arrowed); one clade contains taxa with straight spores (blue box) and the other contains those with curved spores (green box). Figures at the nodes represent percentage bootstrap support values from 1000 samplings. *Cystodiscus melleni* is used as the outgroup and to root the tree.

A more focused analysis of all known sphaeromyxid SSU rDNA sequences, including those with short sequence reads, revealed a robust group divided into strongly supported sub-clades representing those with straight spores or those with curved ones (Figure 9). *Sphaeromyxa lycodi* forms a clade with *S. kenti*, in the group with curved spores with isolates of *S. hellandi*.

The only sequence from non-fish host that grouped outside the hepatic biliary clade was *Chloromyxum careni* isolated from the kidney of the Malayan horned frog *Megophrys nasuta*[22].

## Discussion

To date approximately 40 *Sphaeromyxa* species have been reported, all of which are parasitic in the hepatic biliary systems of marine fishes, typically found in the gall bladder. On the basis of the morphological features of mature spores, they have been divided into two main groups; having either arcuate or straight spores [23], and DNA analysis in the present paper, based on available *Sphaeromyxa* sequences, supports this grouping (Figure 9). Sphaeromyxids have been shown to have unusually low host specificity [24], a characteristic also demonstrated in this study with 5 of the 6 *Lycodes* spp. as hosts. As DNA data for the group is limited (six species now have SSU rDNA data, Figure 9), most descriptions of sphaeromyxids have been based exclusively on morphological characteristics, and therefore, it is important to demonstrate that potential new species are novel. The arcuate species *S. hellandi* and *S. kenti*[8,25] are the most phylogenetically related to *S. lycodi* n. sp., but are too distant to be the considered conspecific (Figure 9). Five other known arcuate species, *S. arcuata*, *S. curvula*, *S. sabrazesi*, *S. elegini* and *S. noblei*, show some resemblance to *S. lycodi* with regard to spore size. However, when compared they are all quite different with respect to one or more features (Table 1). Firstly, all these species have very different geographic distributions with one reported from the Mediterranean, one from Australian waters, one from Japan and Barents Sea and one from the South Atlantic [23,24,26]. With regard to morphology, *S. arcuata* and *S. curvula*, have considerably more slender polar capsules than *S. lycodi*. Furthermore, *S. arcuata* is generally slightly longer while *S. curvula* is slightly shorter than *S. lycodi*[23]. Both the spore body and the polar capsules of *S. sabrazesi* are significantly narrower in addition to the polar filaments being only half the length of those from *S. lycodi*[23]. *Sphaeromyxa elegini* is different in having a very small (10 × 18 μm) disporous plasmodia but also a differently arranged nuclei [26]. Finally, *S. noblei* has a leaf-like plasmodia and differently arranged nuclei [24]. Scanning electron microscopy of spores of *S. lycodi* has allowed us to visualise the shape and arrangement of the two valves (Figures 5 and 6), which appear to be somewhat similar to those in the phylogenetically related species *S. kenti*. This valvular arrangement, one rounded end and one cupped end, may be a common feature in arcuate spore forms but SEM data is limited for the group.

**Table 1:** **Comparison of *S. lycodi *with other arcuate *Sphaeromyxa *species which overlap with regard to spore length**[8,23,25,26]

	**Body**			**Polar capsules**			**Polar filaments Length**	**Plasmodium Type**	**Nuclei Location**	**Geograpic distribution**
	**Length**	**Width**	**W:L**	**Length**	**Width**	**W:L**				
***S. lycodi***	19.6-25.3	4.6-6.9	0.25	6.3-9.8	2.5-4.5	0.40	28.8-48.3	Polysporous Long and slender	Parallel to short axis	North off Iceland
***S. hellandi *Auerbach, 1909**	22.5-30	4.5-7.5	0.21	8.5-12.5	2.5-3.5	0.28	n.d.	Polysporous Leaf like	Parallel to long axis	Atlantic Ocean Barents Sea
***S. kenti *Whipps & Font, 2012**	17.5-19.5	3.8-5.2	0.24	5.8-8.6	2.0-2.6	0.31	n.d.	Polysporous Discoid	Parallel to long axis	Lake Pontchartrain, Louisiana
***S. noblei *Lom, 2004**	18.5-21.5	5.2-6.0	0.28	5.0-6.5	2.5-2.7	0.44	n.d.	Polysporous Leaf like	Parallel to long axis	Australian waters
***S. elegini *Dogiel, 1948**	21-26	4.5-8.8	0.28	7.5-9.0	3.0-4.5	0.45	n.d.	Disporous Small (10x18μm)	Parallel to long axis	Japan Sea Bering Sea
***S. curvula *Fantham, 1930**	19-22	4-6	0,24	7-9	2-3	0.31	n.d.	n.d.	n.d.	S-Atl. Ocean off Namibia
***S. sabrazesi *Leveran et Mesnil, 1900**	22-25	3-4	0,15	8-10	2-3	0.28	12	Polysporous Discoid	n.d.	Mediterranean Sea off Monaco

All measurements in μm. n.d. = no data.

Although *L. reticulatus* and *L. eudipleurostictus*, the type hosts for *S. lycodi*, occupy the same genus, they are readily phylogenetically distinguished from each other using both morphological (tail length) and DNA sequence data [27]. *Lycodes reticulatus* forms part of the monophyletic short tail group that is also supported in multiple gene phylogenies, whilst *L. eudipleurostictus* is part of the sister clade of long-tailed species [27]. We have demonstrated that fish from both major groups of *Lycodes* are host to *S. lycodi*, with the exception of *L. esmarkii*. *Lycodes esmarkii* is a member of the clade of long-tailed species, and hence may also be a host for *S. lycodi*, but was not detected in this study. It is also possible that other related fish genera could be susceptible to infection with *S. lycodi*. Indeed, other sphaeromyxids such as *S. hellandi* and the type species *S. balbianii* are known from multiple fish hosts, often distantly related [24], indicating that sphaeromyxids are not routinely host specific and susceptibility to infection may be due to other factors. The prevalence of infection in adult *Lycodes* spp. was high in most cases, and apart from opacity in some gall bladders no pathology was apparent, suggesting that *S. lycodi* is not pathogenic to the host.

Myxosporeans tend to have a characteristic size of SSU rDNA depending whether they are marine or freshwater species and typically form reliable freshwater and marine clades in phylogenetic analyses [9]. The length of the SSU rDNA sequence for *S. lycodi* is comparable to that of other sphaeromyxids, which are more similar to freshwater myxosporeans than marine species [9]. Indeed, in all of our phylogenetic analyses, the sphaeromyxids are robustly supported in a discrete clade of myxosporeans that infect the hepatic biliary systems of numerous freshwater associated hosts, including fishes, turtles, amphibians, waterfowl and terrestrial insectivorous mammals. However, sphaeromyxids are all described from marine fish, including those from deep water environments such as the *Lycodes* in this study. Our phylogenetic analyses also demonstrate that sphaeromyxids form a well-supported monophyletic group within this freshwater clade and share a common ancestor with *Myxidium coryphaenoideum*. *Myxidium coryphaenoideum* and morphologically similar species *M. melanostigum*, *M. melanocetum* and *M. bajacalifornium* all share numerous characteristics with sphaeromyxids. They are elongate spindle-like myxosporeans with two nuclei; all are known to develop large polysporous plasmodia in the gall bladders of deep sea fish, some with ‘heavy’ polar filaments [11]. *Myxidium coryphaenoideum* is also known to exhibit low host specificity and have an atypical ‘rough’ polar filament [28,29]. These similarities to *Sphaeromyxa* spp. support this type of ancestral *Myxidium* as the correct morphotype for the sphaeromyxids. Fiala [9] supplied the SSU rDNA sequence for *M. coryphaenoideum* and in his phylogenetic analyses he also found it grouped basally to sequences for sphaeromyxids and formed part of a clade of myxosporeans infecting the gall bladders of freshwater fishes. Fiala [9] concluded that sphaeromyxids are closely related to *Myxidium* species, *M. coryphaenoideum* being the closest species and suggested that the common ancestor of marine *Sphaeromyxa* spp. was a freshwater myxosporean with *Myxidium*-shaped spores. We agree that the evidence, both morphological and molecular, is highly indicative that all sphaeromyxids evolved from a common ancestor with an elongate spindle form, similar to that of *M. coryphaenoideum* and the DNA data is supportive of a freshwater origin. However, what is less clear is whether this spore form is the ancestral morphotype for the well-supported hepatic biliary clade (Figures 7 and 8). It has been well reported that *Myxidium* and *Zschokkella*-shaped spores are polyphyletically distributed within myxosporean systematics and hence are assumed to have evolved on numerous occasions throughout myxosporean evolution [9,30,31]. It may be possible that all myxosporean spore forms are as plastic as *Myxidium* over evolutionary time, or it may be that some forms evolve at slower rates and are more likely to be true ancestral morphotypes for clades such as the hepatic biliary group. The majority of known taxa in the hepatic biliary clade (*Myxidium*, *Zschokkella*, *Sphaeromyxa*, *Cystodiscus* and *Soricimyxum*) could have, and likely did, all evolve from an immediate *Myxidium* morphotype ancestry. However, the unambiguous inclusion of *Chloromyxum trijugum* in the group makes it unlikely that the ancestral morphotype for the hepatic biliary clade was *Myxidium*-like. A study of the history of character evolution in myxozoans also indicates that the *Chloromyxum* spore morphotype was more stable during the evolution of the myxosporeans and was responsible for the radiation of freshwater myxosporeans after separation from the marine *Chloromyxum leydigi* group [31] that infects various elasmobranchs (Figure 7). Therefore, we consider it more likely that the ancestral spore form for the hepatic biliary clade was a *Chloromyxum* morphotype.

Myxosporeans from the hepatic biliary clade infecting gall bladders of invasive amphibians, such as the cane toad (*Bufo marinus*) in Australia, are known to spread to numerous endemic species [32,33], again indicating the very low host specificity found in this group of myxosporeans. The spread of wildlife pathogens into new geographical ranges or populations is a conservation concern for endangered species of which amphibian decline is one of the most dramatic examples.

Myxosporeans that infect certain organs or tissues have been shown to reproducibly cluster together in molecular phylogenetic analyses [9,34] and all taxa in the hepatic biliary group are found infecting the gall bladder, bile ducts or liver of their hosts. However, *Myxidium scripta* and *M. hardella*, infecting freshwater turtles, are also reported from renal tubules as well as from the bile ducts and gall bladder [35,36]. This may be due to these reports being from systemic infections, as in both cases severe pathologies and mortalities had occurred, and it is possible that the hepatic biliary system is the initial site of infection with other organs only becoming infected during the advanced stages of infection. However, it may also be possible that turtle myxosporeans in this clade are an exception to this pattern. Currently only a single taxon, *Chloromyxum careni,* isolated from kidney tissues alone, from a non-fish host groups outside the hepatic biliary clade. In our analyses it forms a moderately supported group with other species of *Chloromyxum* that infect the gall bladders of freshwater fishes (Figure 7). However, in other analyses that are more focused on the phylogenetic relationships amongst *Chloromyxum* spp., its position is unresolved and it forms a solitary branch between a clade of urinary bladder infecting species and the *Myxidium lieberkuehni* clade, both of which contain *Chloromyxum* taxa [37]. It is likely that, when more molecular data exist for myxosporean taxa from the renal systems of amphibians, they will form a clade with *C. careni* reinforcing the potential importance of the *Chloromyxum* morphotype as ancestral forms to some of the currently recognised clades in myxosporean systematics.

The sphaeromyxids are currently classified in a separate suborder, the Sphaeromyxina Lom et Noble, 1984, due to the presence of the unique ribbon-like polar filament they all possess. Sphaeromyxids do form a monophyletic clade in this and other phylogenetic studies [8,9], including multiple gene analyses [38] suggesting that this feature is unique amongst the myxosporeans and was derived from an ancestor common to all known sphaeromyxids. However, their assignment to a separate suborder is no longer justified as they are robustly located within taxa from the suborder Variisporina Lom et Noble, 1984, in phylogenetic analyses and have clearly evolved from a common ancestor with an elongate *Myxidium* form, similar to that of *M. coryphaenoideum*. Other recent molecular studies on sphaeromyxids [9,39] and Lom and Dyková’s synopsis of myxozoan genera [40] support these findings; therefore, we recommend that the suborder Sphaeromyxina is no longer retained. However, due to the polyphyletic nature of *Myxidium* in myxosporean systematics, and the placement of the type species, *M. lieberkuehni,* in a different clade to the sphaeromyxids (Figure 7), we retain the family Sphaeromyxidae and place it in the suborder Variisporina Lom et Noble, 1984.

### Taxanomic summary

Phylum: Cnidaria Hatschek, 1888

Unranked subphylum: Myxozoa Grassé 1970

Class: Myxosporea Bütschli, 1881

Order: Bivalvulida Schulman, 1959

Suborder: Variisporina Lom et Noble, 1984

Family: Sphaeromyxidae Lom et Noble, 1984

Genus: *Sphaeromyxa* Thélohan, 1892

### Amended description for genus *Sphaeromyxa*

Polar filament is not tube-like as in other Myxosporea, being flat with a broad base that gradually tapers to the end. In the polar capsule it is irregularly folded several times instead of being spirally wound. Polysporous plasmodia containing disporic pansporoblasts are coelozoic in the gall bladder and bile ducts of marine fishes, and may be several mm in length or diameter. Spore elongated, sometimes slightly curved or arcuate; the two polar capsules lie in its opposite, tapering and truncate ends. Spores open at the level of the straight or curved suture line, bisecting the spore and connecting both its ends. Shell valves smooth or ridged. One binucleate sporoplasm. Marked pathology may result in forms that infect bile ducts.

Main amendments include: polar filament no longer described as short, and pathology may result from infection.

### Specific diagnosis of *Sphaeromyxum lycodi* n. sp

Large polysporous plasmodia, up to 10 mm in length, containing disporic pansporoblasts, are coelozoic in the gall bladder of *Lycodes* spp. In frontal view the spore body is arcuate and tapers towards its rounded ends. In sutural view the spores are slightly sigmoid, tapering somewhat towards the blunt ends. The suture line is relatively indistinct and similar in appearance to the valve striations. Each valve has one bulbous / rounded end that supports the polar capsule with the other end more spoon-like to receive the polar capsule from the opposing rounded valve end. The valves have an almost 180° twist along the suture length so that like ends appear to be in the same plane; there are 6 – 7 striations present on each valve. The pyriform polar capsules encase relatively long and irregularly folded polar filaments. When extruded, the filaments appear flat along their entire length, broad where they exit the spore valve and gradually taper along their length. The sporoplasm is granular with a pair of ovoid and centrally located nuclei.

Type host: *Lycodes reticulatus* and *L. eudipleurostictus*

Type location: Northern Icelandic waters

Site of infection: Coelozoic in gall bladder

Etymology: *lycodi* refers to the generic name of the host fish *Lycodes*

Type material: Deposited at Natural History Museum London (NHMUK) 2013.1 Slide 1 Holotype, 2013.2 Slide 2 Paratype, 2013.3 Slide 3 Paratype.

A SSU rDNA sequence was submitted to Genbank under the accession number KC524734

## Conclusions

*Sphaeromyxa lycodi* n. sp. is a common gall bladder myxosporean in numerous eelpout of the genus *Lycodes* from Northern Iceland. It has characteristics typical of the genus and forms arcuate spores. The spore valves have one rounded end that supports the polar capsule and one spoon-like end to receive the polar capsule from the opposing rounded valve. Molecular phylogenetic analyses confirm that sphaeromyxids form a monophyletic group, subdivided into straight and arcuate spore forms, within the hepatic biliary clade that infect a wide range of freshwater associated animals. The ancestral spore form for the hepatic biliary clade was probably a *Chloromyxum* morphotype; however, sphaeromyxids have more recently evolved from a *Myxidium* ancestor with a spindle-shaped spore. We recommend that the suborder Sphaeromyxina is suppressed; however, we retain the family Sphaeromyxidae and place it in the suborder Variisporina.

## Competing interests

The authors declare that they have no competing interests.

## Authors' contributions

ÁK and MF dissected the fish and isolated the myxosporeans. ÁK performed the morphological and histological studies. MF carried out the DNA analyses and the SEM. ÁK and MF jointly wrote the manuscript. Both authors approved the final version of the manuscript.
